# Prevalence of and Risk factors for Stunting among School Children and Adolescents in Abeokuta, Southwest Nigeria

**DOI:** 10.3329/jhpn.v29i4.8452

**Published:** 2011-08

**Authors:** Idowu O. Senbanjo, Kazeem A. Oshikoya, Olumuyiwa O. Odusanya, Olisamedua F. Njokanma

**Affiliations:** ^1^Department of Paediatrics and Child Health; ^2^Department of Community Health and Primary Care, College of Medicine, Lagos State University, PMB 21266, Ikeja, Lagos, Nigeria

**Keywords:** Adolescent, Child, Child nutrition disorders, Community-based studies, Cross-sectional studies, Nutrition disorders, Risk factor, Stunting, Nigeria

## Abstract

Stunting adversely affects the physical and mental outcome of children. The objectives of the study were to determine the prevalence of and risk factors associated with stunting among urban school children and adolescents in Abeokuta, Nigeria. Five hundred and seventy children aged 5-19 years were selected using the multi-stage random-sampling technique. Stunting was defined as height-for-age z-score (HAZ) of <-2 standard deviation (SD) of the National Center for Health Statistics reference. Severe stunting was defined as HAZ of <-3 SD. The mean age of the children was 12.2+3.41 years, and 296 (51.5%) were males. Ninety-nine (17.4%) children were stunted. Of the stunted children, 20 (22.2%) were severely stunted. Identified risk factors associated with stunting were attendance of public schools (p<0.001), polygamous family setting (p=0.001), low maternal education (p=0.001), and low social class (p=0.034). Following multivariate analysis with logistic regression, low maternal education (odds ratio=2.4; 95% confidence interval 1.20-4.9; p=0.015) was the major contributory factor to stunting. Encouraging female education may improve healthcare-seeking behaviour and the use of health services and ultimately reduce stunting and its consequences.

## INTRODUCTION

Stunting is defined as height-for-age z-score (HAZ) of equal to or less than minus two standard deviation (-2 SD) below the mean of a reference standard (1). It is a well-established child-health indicator of chronic malnutrition which reliably gives a picture of the past nutritional history and the prevailing environmental and socioeconomic circumstances (2). Worldwide, 178 million children aged less than five years (under-five children) are stunted with the vast majority in South-central Asia and sub-Saharan Africa (3). In Nigeria, the national prevalence of stunting among under-five children between 2000 and 2006 was 38% (4).

Stunting is a major public-health problem in low and middle-income countries because of its association with increased risk of mortality during childhood (3,5). Apart from causing significant childhood mortality, stunting also leads to significant physical and functional deficits among survivors (1,3,5). According to the latest reports, stunting contributes to 14.5% of annual deaths and 12.6% of disability-adjusted life-years (DALYs) in under-five children (3). Children who are stunted complete fewer years of schooling. This may be due to the fact that stunted children are known to enroll late in school (6), perhaps because they are not grown enough to enroll. It may also be because they drop out earlier. This may lead to fewer years of education of stunted children when compared with tall children. Stunting hinders cognitive growth, thereby leading to reduced economic potential. In a study on the effects of nutritional status on primary school achievement score in Kenya, undernourished girls were more likely to score less on achievement tests (7). Stunting is known to be highly prevalent in environments that are characterized by a high prevalence of infectious diseases (8). On the other hand, stunting impairs host immunity, thereby increasing the incidence, severity, and duration of many infectious diseases (9). In countries where malaria infection is endemic, stunting increases the degree to which malaria is associated with severe anaemia causing considerably higher likelihood of mortality due to malaria (9).

The long-term consequences of stunting include short stature, reduced capacity of work, and increased risk of poor reproductive performance (1,3). There is a positive association among stunting, central obesity, and cardio-metabolic disorders (10). The burden of these chronic diseases is daunting as they remain significant causes of morbidity and mortality even in the tropics and subtropics. This could stretch health facilities which are either non-existent or ill-equipped to cope with the yet-to-be resolved problems of undernutrition and infections.

In developing countries, most deaths in children are among the under-five children. As a result, there is extensive literature on under-five children compared to dearth of information on the health of school children. Moreover, children who are stunted are likely to remain stunted into adulthood (11). The objectives of this study were, therefore, to determine the prevalence of and risk factors associated with stunting among school children and adolescents in Abeokuta, southwestern part of Nigeria.

## MATERIALS AND METHODS

### Location

This questionnaire-based, cross-sectional study was carried out in randomly-selected primary and secondary (both public and private) schools in Abeokuta. Abeokuta, located on longitude 7’10'N and latitude 3’26'E and is about 100 km north of Lagos, the capital of Ogun State in southwestern part of Nigeria. It has an estimated population of four million. Abeokuta is predominantly made up of people of the Yoruba tribe but urbanization and industrialization have brought in many other ethnic groups.

### Method of sampling

At the time of the survey, there were 322 schools in Abeokuta (the ratio of public to private primary schools was 1:1 while the ratio of public to private secondary schools was 3:1). Using the multistage random-sampling technique, seven schools—two private primary schools, one public primary school, one private, and three public secondary schools—were selected by balloting. This selection process was adopted because the population of students in public primary schools was higher than that of private primary schools while that of secondary schools was about equal. From each selected school, all grades were studied (primary–Grade 1-6 and junior and senior secondary school–Grade 1-6).

Using the estimated prevalence of stunting of 19.8% by Oninla *et al* (12), the minimum sample-size (n) for the study was calculated as follows:

n=z^2^ p (1-p)÷d^2^x2.

where ‘z’ is the critical value, and in a two-tailed test, it is equal to 1.96, p is the estimated prevalence of stunting, and d is the absolute sampling error that can be tolerated. In this study, it was fixed at 5%. Multiplication by 2 was done for correcting design effect. Therefore, the minimum sample-size was: n=1.96^2^x0.198x(1-0.198)÷0.05^2^=488.

On the day of the study, one arm from each class was selected by balloting. Ballot papers were served to all the children in the selected arm. The ballot papers were blank, except those that were marked with number 1 to 15. After all the students had picked a paper, they were asked to open, and those with number 1 to 15 were selected. Ninety pupils were selected from each of the seven schools.

Each student was interviewed to obtain information on demographic and socioeconomic characteristics of the child's family. The families were assigned to a socioeconomic class using the method (modified) recommended by Oyedeji (13). The parents’ occupation and highest education attained were scored from 1 (highest) to 5 (lowest). The mean score for both parents gives social class falling within the 1-5 range. Those with the mean score of <2 were further reclassified into upper class while those with the mean score of >2 were reclassified into lower social class. For the occupation score, those in upper social class included parents, such as senior public officers, large-scale traders, large-scale farmers, and professionals while lower class included artisans and primary school teachers, peasant farmers, labourers, and the unemployed. For the education score, those with PhD, masters degree, bachelors and higher national diploma (HND) were categorized as upper class while those with ordinary national diploma (OND), national certificate of education (NCE), technical education grade II teaching certificate, junior and senior secondary school certificates, primary school certificate, and those with no formal education were classified as lower social class.

### Measurements

Measurements of height were taken by three student nurses who were trained on the standard procedure of measuring height as described below. The correlation coefficients between the nurses were 0.93, 0.97, and 0.99, and this ensures that the error between the nurses can be taking as minimal. The height was measured using a mobile stadiometer that was specifically made by one of the authors (IOS) and calibrated using a standard tape measure. This was done with the child standing erect without shoes and with the eyes looking horizontally and the feet together on a horizontal level. These measurements were done to the nearest 0.1 cm. Standardization checks on the height boards were done periodically during the study period.

### Definitions

In this study, stunting was defined as HAZ equal to or below minus two standard deviation (-2 SD) of the mean of National Center for Health Statistics (NCHS) standard. Severe stunting was also defined as HAZ equal to or below minus three (-3 SD) of this reference standard (1,4).

### Analysis of data

Data were analyzed using the Epi Info 2002 and the SPSS for Windows software (version 11). The means and standard deviations of height and HAZ were calculated by age-groups and sex. Proportions were calculated for categorical variables, and these were compared using the Pearson's chi-square (χ^2^) test. The prevalence of stunting among groups of students was defined using specific sociodemographic characteristics to identify potential risk factors for stunting. Only risk factors with p value of <0.05 were fed into a multiple regression model to determine contribution of these variables to stunting. The statistical significance was established when the p value was less than 0.05 and when confidence interval did not include unity

**Table 1.** Height, height-for-age z-scores, and prevalence of stunting according to age-group and sex

	Age-group		
Parameter	5-9 years	10-14 years	15-19 years
	(n=147)	(n=245)	(n=178)
Height (cm)			
Male	124.7 (7.53)	141.1 (9.97)	163.5 (9.90*)
Female	123.8 (9.05)	142.8 (11.8)	157.1 (6.27)
Sexes combined	124.2 (8.42)	141.8 (10.8)	160.5 (8.96)
Height-for-age z-scores			
Male	-0.171 (1.13)	-1.227 (1.04)	-1.097 (1.06)
Female	-0.353 (1.42)	-0.954 (1.19)	-0.873 (0.99)
Sexes combined	-0.275 (1.31)	-1.109 (1.12)	-0.991 (1.04)
Stunting			
Male (%)	6.3	20.9	22.3**
Female (%)	15.5	17.9	15.5
Sexes combined (%)	11.6	19.6	19.1

Values are mean±SD unless otherwise stated.

p=0.000 for sex difference;

p<0.05 for age difference in males;

SD=Standard deviation

### Ethical clearance

Ethical approval and clearance were obtained from the Federal Medical Centre Research/Ethics Committee and from the Ogun State Ministry of Education respectively. The teachers, pupils, and parents were well-informed of the scope and extent of the survey, and consents of the parents and pupils were also obtained.

## RESULTS

Of 630 pupils who were selected, 570 (90.5%) completed the study. Forty-nine pupils were excluded based on refusal to participate; three had severe bowing of the legs; two had features suggestive of poliomyelitis; and six had features suggestive of sickle-cell disease. Their age ranged from five years to 19 years while their mean age was 12.2+3.41 years. Two hundred and ninety-six (51.5%) were male. The social distribution showed that 165 (28.9%) and 405 (71.1%) children belonged to the upper and lower class respectively.

Table 1 shows the mean height, HAZ, and prevalence of stunting according to age-group and sex. The height increased with age in both males and females (r=0.89, p=0.000; r=0.87, p=0.000). However, as the age increased, there was a decrease in the mean HAZ, getting to a nadir at the age of 12 years in females and 13 years in males (Fig.). Among children aged 15-19 years, the mean height was significantly (p=0.000) higher in males than females.

Ninety-nine (17%) children were stunted with no significant difference between sexes (χ^2^=0.33, p=0.567). These children—nine (3.0%) males and 11 (4.0%) females were—severely stunted. This gave an overall prevalence rate of severe stunting to be 3.5%. The prevalence of stunting increased with age and significantly more so with males (χ^2^=7.67, p=0.022).

**Fig.**
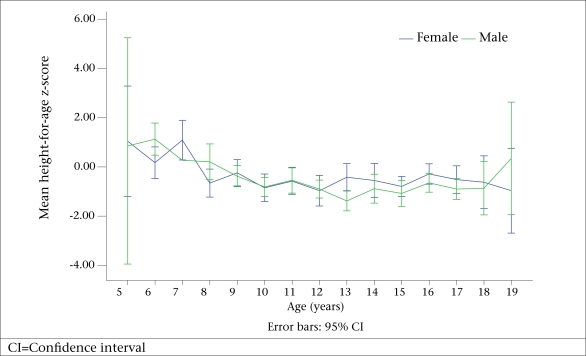
Distribution of mean height-for-age z-score according to age and sex

Table 2 shows the association between stunting and various demographic and socioeconomic factors. The prevalence of stunting was significantly higher among children aged above 10 years (p=0.031), children from polygamous homes (p=0.001), children attending public schools (p=0.000), children of mothers and fathers with low level of education (p=0.0000, p=0.026 respectively), and low social class (p=0.019). These were entered into a multiple regression model as independent variables with stunting as the dependent variable. Following this multivariate analysis (Table 3)., only low level of education of mothers (odds ratio=2.4; 95% confidence interval 1.20-4.9; p=0.015) significantly contributed to the occurrence of stunting in the population studied.

## DISCUSSION

The overall prevalence of stunting in this study was 17.4%. This is similar to the finding of 19.8% among urban school children in Ile-Ife, Nigeria (12), 16.64% among Kenyan middle-school children (7), and 17.9% among Santal children of Puruliya district in India (14). It is lower than the range of 48-56% obtained for children from countries under Partnership for Child Development (Ghana, Tanzania, Indonesia, India, and Viet Nam) (15) while it is high when compared with Turkish children where only 5.7% are stunted (16). This difference is likely to stem from differential nutritional intake, socioeconomic and cultural differences rather than differences in their genetic potential to achieve maximum height. Although the NCHS standard used in this study represents the growth pattern of children from the USA, it is accepted internationally to adequately reflect variation in growth that is related to nutrition and health of children from different ethnic backgrounds (17). The new multi-centre growth reference chart of the World Health Organization (WHO) was not used because there are very few studies that used it on school children and adolescents, and this will make it difficult for us to compare our findings with those of many other studies that used the NCHS. Moreover, Stephenson *et al*. showed that the height of well-off, urban school children in Kenya was not different from the NCHS reference values (15) while Jane *et al*. found that the growth of the ‘elite’ Nigerian children is very similar to the figures of the NCHS (18). Thus, our finding is a true mirror-image of overall standard of living in Abeokuta and capability of the population to meet its basic needs, such as access to food, housing, and healthcare, which themselves are factors closely linked to the development of undernutrition. However, there is likelihood of underestimation of the burden of stunting in this study as our survey was only on school-goers. Results of studies in Ghana and Tanzania showed that non-enrolled children were more undernourished than children enrolled in school (19,20). The rate of enrollment of children in school is very low in Nigeria. Between 2000 and 2006, the net enrollment ratio for Nigerian primary and secondary schools ranged from 25 to 72 (4). Therefore, the prevalence of 17% may just be a tip of the burden of stunting in the whole school-age population.

**Table 2.** Demographic and socioeconomic determinants of stunting

	Nutritional status					
Parameter	Stunted (n=99)		Not stunted (n=471)		Odds ratio	95% CI
	No.	%	No.	%		
Age (years)				
≥10	82	19.4	341	80.6		
<10	17	11.6	130	88.4	1.84	1.02-3.35*
Sex				
Male	54	18.2	242	81.8		
Female	45	16.4	229	83.6	1.14	0.72-1.79
Type of family†				
Polygamy	47	25.0	141	75.0		
Monogamy	51	13.6	323	86.4	2.11	1.32-3.37**
Type of school				
Public	84	21.1	315	78.9		
Private	15	8.8	156	91.2	2.77	1.50-5.19***
Education of mothers				
≦Secondary	75	22.3	262	77.7		
Post-secondary	24	10.3	209	89.7	2.49	1.52-4.09***
Education of fathers				
≦Secondary	62	20.7	237	73.9		
Post-secondary	37	13.7	234	86.3	1.65	1.06-2.58*
Social class				
Lower	80	19.8	325	80.2		
Upper	19	11.5	146	88.5	1.89	1.07-3.36*

†Total number of responses is 562;

*p<0.05;

**p<0.01;

***p<0.0001; CI=Confidence interval

**Table 3.** Logistic regression analysis of risk factors for stunting

Independent variable	Odds ratio	95% CI	p value
Age	0.94	0.68-1.30	0.94
Polygamous family	0.62	0.39-1.00	0.053
Public school attendance	1.89	0.96-3.74	0.07
Low maternal education*	2.39	1.18-4.84	0.015
Low paternal education†	0.68	0.34-1.36	0.28
Low social class	1.02	0.47-2.24	0.96

Dependent variable: Stunting.

Low maternal education; equal to or less than secondary school education;

†Low paternal education; equal to or less than secondary education;

CI=confidence interval

The relationship between stunting and gender varied. While some studies demonstrated a higher prevalence among males (7,12,16), others demonstrated a higher prevalence among females (14,21). In the present study, the prevalence of stunting was higher among young female children aged 5-9 years while the reverse was the case among children aged 15-19 years. This could be due to increased access to food at the older age when the females are culturally involved in the cooking of family-food, and hence, their better nutritional state compared to the male counterparts. Another explanation for the fact that more boys aged 15-19 were stunted than girls could be because poor, stunted girls had dropped out of school leaving behind better-nourished girls. On the other hand, the higher prevalence of stunting among younger female children could have been due to the effect of extension of cultural preference for boys at birth (22,23).

The finding of the higher prevalence of stunting as the age increases is a common occurrence among children in low and middle-income countries where, after the age of three months, there is growth faltering which is persistently low throughout school-age years (15,18). The decline in height compared to the NCHS reference in this study peaked at the age of 12 years in females and 13 years in males, and this is similar to the finding among Zanzibari children (24). This occurrence has been associated with the growth-spurt which either did not occur at all or occur later than is typical in well-nourished children. It is, therefore, important that the nutrient intake during this period must match the requirements for growth, otherwise there will be growth retardation.

Similar to the finding among under-five children by Ojofeitimi *et al.* (25), the prevalence of stunting was higher among children from polygamous homes. Polygamy is a common family-setting among Africans, particularly those from the lower socioeconomic group. It usually has a larger number of people compared to monogamous homes. The larger the number of people in a home, the smaller the amount of food that gets to children, especially in the poorest families (26). There is also the possible risk of overcrowding. This could lead to the spread of diseases, such as acute respiratory infections and diarrhoea which are known to lead to malnutrition. It is, therefore, not surprising that stunting occurred more commonly among children from polygamous families compared to children from monogamous families.

In agreement with most studies (25,27), low maternal education was a major determinant of stunting in the present study. Expectedly, as the level of education of the mother increases, so do her finances and her contribution to the total family-income. This places the family at a higher social class and, therefore, better nutritional status. In addition, mothers who are educated are more likely to make decisions that will improve nutrition and health of their children (28). An educated woman is likely to send all her children to school, thereby breaking the chain of ignorance; she would better use the childhood survival strategies, such as adequate breastfeeding, immunization, oral rehydration therapy, and family planning. Thus, educating women would be a useful step in the reduction of the prevalence of poor childhood nutrition, especially stunting. The association of stunting with attendance of public schools as observed in this study may be a reflection of the socioeconomic status of parents. It is, therefore, important to advocate for the provision of free school-meals for children in such public schools. This would guarantee at least one balanced diet per week-day for these children and improve their growth. Other inexpensive, simple-to-deliver, health promoting and intervention measures, such as periodic treatment for worms, supplementation of micronutrients and participatory health education, should form part of school health services which should be encouraged.

### Conclusions

Chronic malnutrition remains a prominent feature of urban Nigerian school children and adolescents. The United Nations has set Millennium Development Goals (MDGs) to be achieved by 2015 (4). The eight MDGs which include halving the proportion of people suffering from hunger and malnutrition, providing universal primary education, and promotion of higher levels of higher education for both men and women form a blueprint agreed to by all countries of the world. Concerted efforts made to promote higher education in women will help promote women empowerment so that they can be receptive to the use of developmental initiatives.

## ACKNOWLEDGEMENTS

The study was sponsored by the management of Federal Medical Centre, Abeokuta, Nigeria. The authors express their gratitude to the management of Federal Medical Centre, Abeokuta, for sponsoring this work. The authors thank the State Ministry of Education and principals of all schools and teachers for giving them permission to use their pupils. They also thank all the pupils who participated in the study.
